# Use of energy device in general surgical operations: impact on peri-operative outcomes

**DOI:** 10.1186/s12893-022-01540-z

**Published:** 2022-03-09

**Authors:** Olalekan Olasehinde, Afolabi Owojuyigbe, Adekunle Adeyemo, Arinzechukwu Mosanya, Olurotimi Aaron, Funmilola Wuraola, Temitope Owoniya, Temilola Owojuyigbe, Olusegun Alatise, Adewale Adisa

**Affiliations:** 1 Department of Surgery, Obafemi Awolowo University Teaching Hospitals Complex, PMB 5538, Ile-Ife, Nigeria; 2 Department of Anaesthesia, Obafemi Awolowo University Teaching Hospitals Complex, Ile-Ife, Nigeria; 3 Department of Otorhinolaryngology, Obafemi Awolowo University Teaching Hospitals Complex, Ile-Ife, Nigeria; 4 Department of Haematology, Obafemi Awolowo University Teaching Hospitals Complex, Ile- Ife, Nigeria

**Keywords:** Energy, Devices, Ligasure

## Abstract

**Background:**

The introduction of energy devices has significantly expanded the scope of surgical expedition. The LigaSure™ vessel sealing system is a bipolar electrosurgical device, recently introduced to our practice. Its impact on peri-operative outcomes in a variety of major operations was evaluated in this study.

**Methods:**

A retrospective review of operations performed following the adoption of the LigaSure™ vessel sealing device was carried out. Five categories of operations were evaluated (Thyroidectomies, Gastrectomies, Colectomies, Pancreaticoduodenectomies, and Anterior/Abdomino-perineal resection [A/APR of the rectum). Peri-operative outcomes (duration of operation, intra-operative blood loss, blood transfusion rates) were compared with a cohort of similar operations performed using conventional techniques. Data analysis and comparisons were done on a subgroup basis.

**Results:**

A total of 117 operations were performed using the LigaSure™ device with thyroidectomies being the most common (66/117-56.4%). Compared to cases done using conventional techniques of suture and knot with electrocautery (120 cases), the use of LigaSure™ was associated with a significant reduction in operation time in all categories of operations. Intraoperative blood loss was also lower in all categories of cases, but this was only statistically significant following A/APR and Thyroidectomies. Generally, there was a trend towards a reduction in blood transfusion rates.

**Conclusions:**

The use of energy devices for surgical operations is feasible in a resource-limited setting. It has the potential of improving outcomes.

## Introduction

Surgical practice has undergone major refinements with corresponding improvements in post-operative outcomes over the years. Technological solutions such as energy devices are some of the important innovations that have contributed to these improvements. The use of thermal energy for surgical haemostasis began with the invention of the electrosurgical unit by Bovie and later the development of the bipolar diathermy by Dr. Leonard Malis [1, 2]. This opened up new frontiers, with the development of haemostatic devices applying different energy modalities such as ultrasonic, and laser energy [2, 3]. The LigaSure™ Vessel Sealing Device (Medtronic, Minneapolis, MN, USA) is an advanced bipolar electrosurgical device which achieves haemostatic seal by combining bipolar electrocoagulation and pressure, resulting in coagulation of elastin and collagen on the vessel wall [4]. It also incorporates a tissue-based feedback program that regulates precisely, the dosage of applied energy [5]. Its use has been reported in thyroidectomies, haemorrhoidectomies, gynaecological operations and, some abdominal operations [6–11]. The majority of these reports are from Western climes with very limited contribution from sub-Saharan Africa where patient characteristics and surgical outcomes are known to be different. Very little is known about the use of energy devices in sub-Saharan Africa. Given the poorer surgical outcomes in sub-Saharan Africa (SSA), it will be interesting to know what impact the adoption of energy devices has had on surgical outcomes. Following its adoption into our practice, the LigaSure™ device was first used for thyroidectomies, with very promising results after a preliminary review of 30 patients [12]. Its use has since been expanded to include a variety of other major surgical operations. We report our experience with the use of the device and its impact on perioperative outcomes after three years of sustained use in a low- middle-income country.

## Methodology

This was a retrospective review of major surgical operations performed between 2017 and 2019 after the adoption of the LigaSure™ device. A retrospective cohort of similar operations consecutively performed between 2014 and 2016 using conventional haemostatic techniques (sutures and diathermy), before the adoption of the LigaSure™ device were selected for comparison. The operations included thyroidectomies, gastrectomies, colectomies, pancreaticoduodenectomies, and rectal operations (Low/Anterior Resection of the Rectum—LAR, and Abdominoperineal Resection of the Rectum—APR). Case selection was based on the year and type of operation. In both groups, consecutive cases managed during the stipulated periods were included. Regarding the type of operation, the selection was based on the general classification of the operations with no standardization in terms of indication (benign versus malignant), size of the lesion, and extent of resection (Left and right hemicolectomy were all considered as colectomies, subtotal and total gastrectomy were all considered as gastrectomies, while total, sub-total or partial thyroidectomy were all classified as thyroidectomies). The same model of Ligasure device was used all through the period under review (Covidien Valley Lab LS10). The outcome measures were: duration of operation, peri-operative blood loss, and perioperative blood transfusion rates. Duration of operation (skin incision to skin closure) was obtained from the operating record, while intraoperative blood loss was obtained from the anaesthetists’ record of estimated blood loss. Blood transfusion rate was limited to the use of blood products perioperatively as recorded by the anaesthetist. Peri-operative blood transfusion was defined as blood transfused during the operation and in the immediate post-operative period. Comparison was done on a subgroup basis according to the type of operation performed. Data analysis was done using IBM SPSS statistics for windows, version 22 (IBM Corp., Armonk, N.Y., USA.

## Results

In all, 237 operations were analyzed, 127 thyroidectomies, 17 gastrectomies, 58 colectomies, 18 Pancreaticoduodenectomies, and 17 cases of abdominoperineal/Anterior resection of the rectum. There were 117 cases in the LigaSure™ group and 120 cases in the conventional group (Table 1). For each type of operation, patients in the LigaSure™ and conventional groups were similar in terms of age and gender distribution (Table 1). The use of LigaSure™ device was associated with a statistically significant reduction in operation time in all categories of operations ranging from a 19.3% reduction in pancreaticoduodenectomies (429 ± 87 vs. 346 ± 48, p = 0.01) to 38.4% reduction in gastrectomies (273 ± 103 vs. 168.3 ± 64, p = 0.04).

There was also a reduction in intraoperative blood loss ranging from a 20.3% reduction in colectomies (615 ± 408 vs. 490 ± 243, p = 0.2) to 67% reduction in A/APRR. (1592.9 ± 574 vs. 490 ± 243, p = 0.01, Table 1). Reduction in intra-operative blood loss was however only statistically significant in the thyroidectomy and A/APRR subgroups (43.4% reduction, p = 0.001, and a 67% reduction, p = 0.01 respectively). Blood transfusion rates were also generally lower in the LigaSure™ group but none was statistically significant. There was no adverse intraoperative event recorded following the use of the device.

**Table 1:** Patient characteristics and perioperative outcomes

Type of operation	Conventional technique	LigaSure™	% reduction	P value
Thyroidectomy	N (61)	N (66)		
Mean age (years)	43.8 ± 13.5	44.4 ± 13.9		0.8
Sex				
Male	6 (9.8%)	6 (9.1%)		0.45
Female	55 (90.2%)	60 (90%)		
Mean operation time (mins)	118.5 ± 32	72 ± 38	34.1	0.002
Estimated blood loss (mls)	323.4 ± 238	183 ± 218	43.4	0.001
Blood transfusion rate 1 (1.6%)		0		0.3
Gastrectomy	N (11)	N (6)		
Mean age (years)	53.1 ± 11	53.8 ± 7		0.8
Sex				0.6
Male	5 (49.5%)	6 (54.5%)		
Female	8 (47.1%)	9 (52.9%)		
Mean operation time (mins)	273.6 ± 103	168.3 ± 64	38.4	0.04
Estimated blood loss (mls)	875 ± 1147	358 ± 198	40.9	0.2
Blood transfusion rates	7 (63.6%)	4 (66.7%)		0.6
Pancreaticoduodenectomy	N (7)	N (11)		
Mean age (years)	53.7 ± 10	59.1 ± 9.6		0.2
Sex				0.3
Male	3 (42.9%)	7 (63.60%)		
Female	4 (57.1%)	4 (36.40%)		
Mean operation time (mins)	429 ± 87	346 ± 48	19.3	0.01
Estimated blood loss (mls)	1660 ± 1.333	857 ± 660	48.4	0.19
Blood transfusion rates	4 (57.1%)	4 (45.5%)		0.7
Colectomy	N (33)	N (25)		
Mean age (years)	49 ± 14	55 ± 13		0.1
Sex				0.6
Male	20 (60.6%)	13 (52%)		
Female	13 (39.4%)	12 (48%)		
Mean operation time (mins)	222.6 ±68	169.3 ± 68	23.9	0.004
Estimated blood loss (mls)	615 ± 408	490 ± 243	20.3	0.2
Blood transfusion rates	17 (51.40%)	11 (44.40%)		0.2
A/APRR	N (8)	N (9)		
Mean age (years)	53.5 ± 12	49.9 ± 15		0.59
Sex				0.58
Male	3 (37.5%)	4 (44.4%)		
Female	5 (62.5%)	5 (55.6%)		
Mean operation time (mins)	386.4 ± 83	249.6 ± 63	35.4	0.002
Estimated blood loss (mls)	1592.9 ± 574	525 ± 244	67	0.01
Blood transfusion rates	6 (85.7%)	4 (44.4%)		0.07

*A/APR* Anterior/Abdominoperineal resection of the rectum

## Discussion

The use of energy devices for surgical operations has been in practice in many parts of the world for a few decades [6, 13, 14]. Such experience has however been scantily documented in low resource settings probably due to limitations of cost and training. The need to adopt modern technology within the limits of available resources in low-resource settings is however imperative for better surgical outcomes. Often, operations are performed on physiologically compromised patients due to late presentation or advanced disease, justifying the need for quick operations with minimal physiological trespass which can be achieved using energy devices. The limited access to blood products in low-resource settings is an additional incentive to adopt measures that will limit blood loss during operations. These are some of the advantages of the use of energy devices highlighted in this study as in some earlier reports. Our experience demonstrates the feasibility of successfully adopting the use of the device in a low resource setting having routinely deployed it in a variety of operations. Our review also shows that the use of the device was associated with a significant reduction in operation time and a trend towards less intraoperative blood loss and the use of blood products in all the categories of operations analyzed.

The reduction in operation time in the various types of surgical procedures evaluated in this study agrees with existing data. Macario and colleagues in a metanalysis reported a significant reduction in operation time in 24 out of 26 studies on the use of LigaSure™ in different types of operations [6]. Reduced operation time has significant implications for patients, operating staff, and the system in general. Studies evaluating the impact of long operation time on outcomes show clearly that patients with prolonged operation time tend to do worse for a variety of reasons [15–17]. Long operation time is associated with a higher risk of surgical site infections due to prolonged exposure to microorganisms, reduced efficacy of perioperative antibiotics, increased risk of breach of aseptic technique, and the risk of tissue ischemia from prolonged tissue retraction and handling. Increased risk of thromboembolic events due to prolonged stasis and endothelial injuries also contribute to worse outcomes [17]. Cheng et al. [16] in a metanalysis of 66 studies showed a robust association between prolonged operation time and the development of complications across all surgical specialties. They noted that for every 30 min increase in operation time, there is a 14% risk of developing post-operative complications [16]. Reduced operation time also helps to reduce fatigue among members of the operating team and frees up theatre spaces for other operations, thereby reducing case cancellations and delays. These may cumulatively impact positively on post-operative outcomes.

Intra-operative blood loss is an important peri-operative event that has profound effects on patient outcomes [18, 19]. Excessive bleeding compromises patients’ physiological state and also increases the risk of operative injuries and postoperative mortality. The use of LigaSure™ in this study demonstrated a trend towards lower intraoperative blood loss in all types of operations. Although this was only statistically significant following thyroidectomy and rectal operations, the percentage reduction in blood loss with the use of LigaSure™ may be considered clinically significant. While most studies agree on the significant reduction in blood loss associated with the use of LigaSure™ for thyroidectomies [8, 12, 20], outcomes vary for the other types of operation. Tobias et al. [21] in a pilot study compared the use of LigaSure™ during pancreaticoduodenectomy with conventional techniques and showed that the use of LigaSure™ was associated with lower intraoperative blood loss. Eng et al. [14] in a similar study however reported only a reduction in operation time while intraoperative blood loss was similar between the two techniques. Studies on the impact of LigaSure™ on blood loss during gastrectomy and colectomy have also yielded mixed results. Zhou et al. [22] in a matched pair analysis of the short- and long-term outcomes of LigaSure™ versus conventional surgery for curative gastric cancer resection observed that LigaSure™ was associated with shorter operation time and hospital stay, less blood loss compared to conventional surgery. Fujita and colleagues however concluded that Ligasure use did not contribute to reducing intraoperative blood loss or other adverse surgical outcomes when compared to conventional techniques in patients undergoing curative gastric surgeries [23].

The benefits of Ligasure™ in laparoscopic colectomies and rectal procedures are well documented [24, 25], however, there is a paucity of data on its application in open colectomies, as well as for abdominoperineal or anterior resection of the rectum. This study, therefore, provides important data on the usefulness of LigaSure™ for rectal operations demonstrating a significant reduction in both operation time and blood loss.

Adopting the use of energy devices in resource-poor settings comes with some challenges. Chief among these is the issue of cost which limits the procurement of enough devices to service all demands. We adopted an approach in our practice in which elective operations are arranged by various surgical units in a manner that allows the device to be alternated between operating rooms. Except for occasional overlaps, this approach has significantly aided the routine use of the device for all eligible cases. The cost of consumables (handpieces) poses another challenge to its adoption in a resource-limited setting. Encouraging the re-use of handpieces by the manufacturing company, making it more durable for multiple operations will certainly be a huge investment into surgical practice in resource-limited settings. Such innovation has been successfully introduced with the design of re-useable metallic intestinal stapler handles.

An obvious limitation of this study is its retrospective and non-randomized design. Important variables such as comorbidities, lesion size or tumour stage (for malignancies), and other possible confounders were not considered. This study also did not evaluate important post-operative outcomes such as mortality, morbidities, and duration of admission. An initial study that evaluated a few cases of thyroidectomy using the Ligasure device in our institution however demonstrated a significant reduction in duration of hospitalization from 3.5 to 1.9 days with the use of the device [12]. A prospectively designed trial evaluating these important post-operative details in the various categories of operations reported in this study will be ideal.

Within these limits, however, this study shows the versatility and usefulness of the LigaSure™ device in a variety of surgical operations and the potential impact on surgical outcomes in a resource limited setting.

## Conclusions

The use of energy devices for routine surgical practice in a resource-limited setting is feasible with no negative effects. It has potential benefits which need to be further explored.

## Data Availability

The datasets used during the current study are not publicly available as we do not have institutional permission to share publicly. It can however be made available from the corresponding author on reasonable request.
